# Development and Evaluation of Poorly Water-Soluble Celecoxib as Solid Dispersions Containing Nonionic Surfactants Using Fluidized-Bed Granulation

**DOI:** 10.3390/pharmaceutics11030136

**Published:** 2019-03-20

**Authors:** Hyeok Jin Kwon, Eun-Ji Heo, Young-Hwan Kim, Sarah Kim, Young-Ha Hwang, Ji-Mi Byun, Se Hyeop Cheon, Sang Yeob Park, Dong Yun Kim, Kwan Hyung Cho, Han-Joo Maeng, Dong-Jin Jang

**Affiliations:** 1 Department of Pharmaceutical Engineering, Inje University, Gimhae 50834, Korea; anfahs12@oasis.inje.ac.kr (H.J.K.); k75610527@gmail.com (E.-J.H.); awdsnm1819@nate.com (Y.-H.K.); abgc123@naver.com (S.K.); xofla111@naver.com (Y.-H.H.); byunjimi55@naver.com (J.-M.B.); csh950824@naver.com (S.H.C.); 2 Institute of Digital Anti-Aging Healthcare, Inje University, Gimhae 50834, Korea; 3 Samyang Biopharmaceuticals Corporation, Seongnam 13488, Korea; lifenature@naver.com; 4 Industry-Academic Cooperation Foundation, Inje University, Gimhae 50834, Korea; dyoonkim0420@naver.com; 5 College of Pharmacy, Inje University, Gimhae 50834, Korea; chokh@inje.ac.kr; 6 College of Pharmacy, Gachon University, Incheon 21936, Korea

**Keywords:** celecoxib, solid dispersion, nonionic surfactants, Cremophor RH40, fluid-bed granulation, tableting, pharmacokinetics

## Abstract

The purpose of this study is to develop a solid dispersion system with improved dissolution, absorption, and patient compliance of poorly water-soluble celecoxib (CXB). Instead of sodium lauryl sulfate (SLS), an anionic surfactant used in the marketed product (Celebrex^®^), solubilization was performed using non-ionic surfactants with low toxicity. Cremophor RH40 (Cre-RH) was selected as the optimal solubilizer. Granules and tablets containing CXB and Cre-RH were prepared via fluid-bed and tableting processes, respectively. The morphology, crystallinity, flowability, dissolution, and pharmacokinetics for CXB-solid dispersion granules (SDGs) and the hardness and friability for CXB-solid dispersion tablets (SDTs) were evaluated. The solubility of CXB was found to be increased by about 717-fold when using Cre-RH. The dissolution of granules containing Cre-RH was found to be increased greatly compared with CXB API and Celebrex^®^ (66.9% versus 2.3% and 37.2% at 120 min). The improvement of the dissolution was confirmed to be the same as that of granules in tablets. The CXB formulation resulted in 4.6- and 4.9-fold higher AUC_inf_ and *C*_max_ of CXB compared with those of an oral dose of CXB powder in rats. In short, these data suggest that the solid dispersion based on Cre-RH—a non-toxic solubilizer, non-ionic surfactant— may be an effective formulation for CXB to enhance its oral bioavailability and safety.

## 1. Introduction

Among the various pharmaceutical formulations, solid dispersion is known to be one of the most effective strategies to improve the solubility, dissolution rate, and oral bioavailability of poorly water-soluble drugs [1,2]. Solid dispersion shows a reduction in particle size and an increase in surface area of poorly water-soluble drugs, resulting in improved solubility, dissolution rate, and oral bioavailability [3,4]. Solid dispersion is often produced by co-precipitation, co-evaporation, or co-grinding methods [5,6]. However, these methods produce very poor physical properties, such as flowability, mixing properties, and compressibility, due to the semi-solid excipients used together in dosage form preparations [7], which makes them unsuitable for producing commercial products in large quantities [8]. Recently, a solid dispersion preparation method using a fluid-bed granulation process has been attracting attention as a manufacturing method capable of overcoming such drawbacks. The fluid-bed granulator can perform several unit operations within the same equipment continuously, such as pre-blending, granulation, and drying [9], and the granules produced in this process are processed by undergoing tableting and filling processes [10], which are favorable for scale-up for commercialization [11].

Celecoxib (CXB), selective COX-2 inhibitor, and non-steroidal anti-inflammatory drugs are frequently prescribed for the treatment of pain, acute pain resulting from inflammation, rheumatoid arthritis, osteoarthritis, and ankylosing spondylitis [12,13,14]. Although CXB has a variety of therapeutic efficacies, it is apparent that its dissolution rate and oral bioavailability are very poor due to its extremely low aqueous solubility (about 3 μg/mL) [15,16]. CXB’s marketed product (Celebrex^®^ manufactured by Pfizer) is designed to significantly improve solubility and oral absorption through the micronization of CXB particles and the use of a solubilizer [17,18]. Sodium lauryl sulfate (SLS), an anionic surfactant used as a solubilizer in Celebrex^®^, has a very strong solubilizing ability but is also known as a toxic substance that causes strong mucosal irritation [19,20]. In addition, ionic surfactants can cause structural modification of proteins and phospholipids and misfunction of phospholipids and enzymes, which result in toxic symptoms in organs and human systems [21]. Therefore, there is a need to develop dosage forms that can improve the oral bioavailability of CXB without the use of highly toxic ionic surfactants [22,23]. Again, the use of non-ionic surfactants, which have advantages over ionic surfactants in terms of toxicity, mucosal compatibility, and biodegradability, may be considered [20,24]. In addition, the development of improved dosage formulations of CXB, a high-dose drug (generally 200–400 mg twice daily), will be able to improve oral bioavailability and thus reduce toxicity and enhance patient compliance [25]. Various formulations of CXB, such as a self-emulsifying system [26], mesoporous silica [27], nanosuspension [25,28], elixir [29], and solid dispersion [30,31] have been developed with respect to the issues of disadvantage and inconvenience. In general, self-emulsifying and nanosuspension systems are liquid dosage forms, and dry elixir systems contain a considerable amount of ethanol in the dosage form. After undergoing several processes for manufacturing a mesoporous structure, the mesoporous silica system also requires additional steps to load the drug solution into the vehicles. Among these, solid dispersion is a very advantageous method because of its easy production, high solubilization, and stabilization effect [32,33]. To improve the solubility and oral absorption of CXB, several studies have been reported for solid dispersion systems using solubilizing agents such as Gelucire, Poloxamer, Ryoto ester, TPGS, and phosphatidylcholine [30,31,34,35]. These reports do not include the preparation of granules and tablets for the commercial production of CXB formulation.

Here, we attempted to develop and evaluate a granule and tablet formulation based on solid dispersion with non-ionic surfactants instead of toxic anionic surfactants used in the marketed products to enhance the solubility, dissolution, and oral bioavailability of CXB. To the best of our knowledge, there has been no study that has tried to improve the dissolution rate and pharmacokinetic profile of CXB by tablet formulation based on solid dispersion with a non-toxic, non-ionic surfactant. We utilized a fluid-bed granulator and a compression machine for the granulation and tableting process and used the appropriate non-ionic surfactant selected from the solubility test as the solubilizer. The morphology, crystallinity, flowability, dissolution, and pharmacokinetic profiles of CXB, solid dispersion or tablet, were carefully evaluated in terms of industrial applicability.

## 2. Materials and Methods

### 2.1. Materials

CXB and SPAN 80 were purchased from Cadila Pharmaceutical Ltd. (Ahmedabad, India) and Sigma Chemical Co. (St. Louis, MO, USA), respectively. Cremophor RH40 (Cre-RH), Poloxamer 188, Poloxamer 407, and Soluplus were received as gift samples from BASF (Ludwigshafen, Germany). Labrafac PG, Labrafil M 1944 CS, Labrasol, Plurol Oleique CC497, and Transcutol HP were obtained from Gattefosse (St Priest, France). Ryoto sugar ester L-1695 and P-1670 were kindly provided by Mitsubishi-Kasei Chemical Co. (Osaka, Japan). Heweten 101 (microcrystalline cellulose) and Pharmatose 200M (Lactose) were acquired from JRS Pharma (Rosenberg, Germany) and DMV-Fonterra Excipients (Goch, Germany), respectively. All other chemicals were of reagent grade.

### 2.2. Determination of CXB Solubility in Solution with Solubilizer

The solubilities of CXB in aqueous solutions with solubilizers (Cre-RH, Labrafac PG, Labrafil M 1944 CS, Labrasol, Plurol Oleique CC497, Poloxamer 188, Poloxamer 407, Ryoto sugar ester L-1695, Ryoto sugar ester P-1670, Soluplus, SPAN 80, or Transcutol HP) were determined. After adding an excess of CXB into 1 mL solutions with solubilizers (5%, *w*/*w*) in tubes, the mixtures were shaken by a rotating mixer at room temperature for 48 h. After equilibrium, the tubes were centrifuged at 3000 rpm for 10 min, and excess insoluble CXB powder was removed by syringe filtration. The solubilized CXB contents in the solutions were quantified using an HPLC system (Agilent 1100 System, Santa Clara, CA, USA) equipped with a UV detector (Agilent, Santa Clara, CA, USA) and reverse-phase C_18_ column (Aegispak; 5 μm, 4.6 × 150 mm; Seongnam, Korea). The mobile phase consisted of distilled water and methanol (25:75, *v*/*v*) and a flow rate of 1.25 mL/min. Chromatograms were monitored at wavelength of 250 nm.

### 2.3. Preparation of Solid Dispersion and Tablets

Solid dispersion granules (SDGs) were prepared using a top-spray technique in an Enger fluid-bed granulator (Chongqing, China). Cre-RH (1, 2, and 5 g) were dissolved in 250 mL of an aqueous solution (with 2.5 g HPMC) at 50 °C. Then, 10 g of CXB powder was slowly added to the solution and stirred for several hours to obtain a homogenous state. The resulting homogeneous solution was sprayed under appropriate conditions on a flowing mixture (microcrystalline cellulose 60 g, lactose 30 g, crospovidone 3 g, Aerosil 200 1.5 g, and talc 3 g) in a fluid-bed granulator. Three types of granules (SDG (CXB/Cre-RH = 1:0.1), SDG (CXB/Cre-RH = 1:0.25), SDG (CXB/Cre-RH = 1:0.5)) were prepared according to the amount of Cre-RH. The process parameters of the granulation were as follows: inlet air temperature, 70–80 °C; product temperature, 25–35 °C; and atomization pressure, 2.0–3.0 bar. 

After the formation of granules in a fluid-bed granulator, an additional drying process was carried out for 20 min at an inlet air temperature of 50 °C. Magnesium stearate (1%, *w*/*w*) was added to the obtained SDGs for lubrication efficacy, and the powder containing the SDGs was passed through a sieve to remove coarse particles. The obtained mixture was mixed in a V-type mixer for 5 min at 30 rpm and then compressed into tablets (equivalent to 50 mg CXB) at a compression level of 7.0–8.0 (about 1000–1500 Kg/cm^2^) using a single-type Riva SA Minipress MII tableting machine (Buenos Aires, Argentina) equipped with 11.0 mm round punches. 

### 2.4. Scanning Electron Microscopy (SEM)

The morphologies of CXB powder and the SDGs were examined by Hitachi S-4300 SE SEM (Tokyo, Japan). Each sample was mounted onto a carbon film and sputtered with platinum before observation; the voltage was set at 15 kV.

### 2.5. Differential Scanning Calorimetry (DSC)

The solid-state characterization of CXB, physical mixture with excipients, physical mixture with excipients, and CXB and SDGs were analyzed by a TA Q20 DSC system (Leatherhead, UK). Each sample (approximately 5 mg) was weighed in an aluminum pan and crimped with a lid. The obtained cans were scanned from 40 °C to 200 °C at a heating rate of 10 °C/min under a dry nitrogen constant flow rate of 20 mL/min.

### 2.6. Flowability of SDG

Flowability of the prepared SDGs was evaluated by the Hausner ratio and Carr’s index calculated from bulk density and tapped density. Bulk and tapped densities were determined using a 20 mL graduated cylinder. The bulk density was measured by carefully pouring the granules into a cylinder and was calculated by dividing the weight of the granule by the volume in the cylinder. The cylinder was tapped from a height of 5 cm until a constant volume. Loose bulk density (LBD) and tapped bulk density (TBD) were calculated from measured bulk volume and tapped volume. The equations used to obtain the Hausner ratio and Carr’s index are shown below.

LBD = SDG weight/bulk SDG volume(1)

TBD = SDG weight/tapped SDG volume(2)

Hausner ratio = TBD/LBD(3)

Carr index (%) = [(TBD − LBD) × 100]/TBD(4)

### 2.7. Hardness and Friability of SDG Tablet

The hardness and friability of solid dispersion tablets (SDTs) were measured using a Monsanto MHT-20 tablet hardness tester (Campbell, Mumbai, India) and a Labfine friability tester (Anyang, Korea). For each measurement, more than 10 tablets were used.

Friability (%) = (*W*_1_ − *W*_2_)/*W*_1_ × 100 (5)

where *W*_1_ is the total tablet weight and *W*_2_ is the total tablet weight after the friability test.

### 2.8. Dissolution Study of SDGs and SDG Tablets

A dissolution study of CXB, Celebrex^®^ (marketed product), and SDGs (CXB/Cre-RH = 1:0.1, 1:0.25, and 1:0.5) was conducted using the paddle method described in the United States Pharmacopeia 24 (Apparatus II). The experiment was performed at 37.0 ± 0.5 °C with a paddle speed of 50 rpm. Samples containing an amount equivalent to 3.0 mg CXB powder were uniformly dispersed in the dissolution media (900 mL solution of pH 1.2 without SLS). The mixture powder contained in the hard gelatin capsules (Celebrex^®^) was taken out and weighed, and an amount equivalent to 3.0 mg CXB was exactly taken out and used in the dissolution test. When the dissolution test was started, some of the powders were observed to be floating on the media surface, but soon they were completely dispersed in the media. Samples (3 mL) of each medium were obtained at predetermined times (5, 10, 15, 30, 45, 60, 90, and 120 min) and replaced by an equal volume of dissolution medium. The collected samples were filtered using a syringe filter (0.45 μm) and analyzed by HPLC as described in Section 2.2. Dissolution tests for the comparison of SDGs and SDTs were also performed. Unlike the tests of CXB powder, Celebrex^®^, and SDGs (equivalent to 3.0 mg CXB), the dissolution tests for the comparison of SDGs and SDTs (both formulations containing 50.0 mg CXB) were conducted in 900 mL pH 1.2 solutions added with 0.5% SLS to maintain sink conditions due to the extremely low aqueous solubility of CXB (about 3.0 µg/mL).

### 2.9. In Vivo Oral Pharmacokinetic Studies in Rats 

To investigate the possibility of enhanced absorption for the optimized SDG (CXB/Cre-RH = 1:0.5) in vivo, oral pharmacokinetic studies of celecoxib powders, a celecoxib marketed product, and the SDGs were performed at a dose of 5 mg/kg in fasted male Sprague Dawley (SD) rats. All animal experiments were performed in accordance with the Guidelines for Animal Care and Use issued by Gachon University, as described previously [36]. Experimental protocols regarding the animals used in this study were reviewed and approved by the Animal Care and Use Committee of the Gachon University (#GIACUC-R2018004, approval date on 11 May 2018). SD rats weighing 260–280 g were purchased from Orient Bio (Seongnam, Korea). The SD rats were fasted overnight but allowed free access to water. Under anesthetic conditions with Zoletil (20 mg/kg, intramuscular injection), the femoral artery was cannulated with a polyethylene tube (PE-50; Clay Adams, Parsippany, NJ, USA) filled with heparinized saline (20 IU/mL), which prevents blood clotting during blood collection. After recovery from surgery, the rats were administered a single oral dose of 5 mg/kg of celecoxib for the celecoxib powders, a celecoxib marketed product, and the SDGs group. At predetermined timepoints (0 (blank), 15, 30, 60, 120, 180, 240, 360, 480, 1440, and 2880 min after the drug administration), ~100 μL of blood samples were collected from the femoral artery cannula. Upon centrifuging whole blood at 12,000× *g* at 4 °C for 10 min, plasma fraction was obtained, and then, the samples were stored at −20 °C until bioanalysis by LC–MS/MS. For the sample preparation, a simple deproteinization method was applied with 100 μL of methanolic internal standard solution (atorvastatin, 500 ng/mL). After quick vortex-mixing and centrifugation at 12,000× *g* at 4 °C for 15 min, the supernatant was obtained for analysis. To determine the concentration levels of Celecoxib, a LC–MS/MS bioanalytical method was applied with our previously developed method (Kim et al., 2018). Briefly, the LC–MS/MS system consisted of an Agilent HPLC system (1290 Infinity, Agilent Technologies, Santa Clara, CA, USA) and Agilent 6490 QQQ mass spectrometer with a positive electrospray ionization (ESI+) Agilent Jet Stream ion source (Agilent Technologies, Santa Clara, CA, USA). To achieve a good separation of celecoxib and Atorvastatin (IS) from the endogenous plasma substances, Synergi 4 μm polar-RP 80A column (150 mm × 2.0 mm, 4 μm, Phenomenex, Torrance, CA, USA) was used using the mobile phase of 0.1% formic acid and methanol (65:35, *v*/*v*) at a flow rate of 0.2 mL/min. Multiple reaction monitoring (MRM) in the ESI+ mode was selected as follows: celecoxib, 381.9 → 362.0; IS (atorvastatin), 559.2 → 440.2. The data was acquired using the Mass Hunter software (version A.02.00; Agilent Technology, Santa Clara, CA, USA). The calibration curves for celecoxib were linear over the range from 1 to 10,000 ng/mL (correlation coefficient *r*^2^ > 0.990). The limit of detection (LOD) and the lower limit of quantitation (LLOQ) were experimentally observed to be 0.2 ng/mL and 1 ng/mL, respectively.

The peak concentration (*C*_max_) and time to reach *C*_max_ (*T*_max_) were read directly from the plasma concentration–time profiles observed. Non-compartmental analysis using Winnonlin 5.0.1 (Pharsight, Cary, NC, USA) was performed to calculate other pharmacokinetic parameters, as described previously [25]. Finally, the relative oral bioavailability (BA) was calculated by dividing AUC after the oral administration of the optimized SDG or the marketed product by that of the celecoxib powder group. To indicate statistically significant differences, a *p*-value of ≤0.05 was used based on a t-test between two means for unpaired data or a Duncan’s multiple range test posteriori analysis of variance (ANOVA) among three means for unpaired data. All data are expressed as mean ± standard deviation (SD).

## 3. Results

### 3.1. Selection of an Optimal Solubilizer for Solid Dispersion System

To find an appropriate solubilizer, the saturated solubility of CXB was measured in various oils and surfactants (Table 1). The experimental results confirmed that the used solubilizers increased the solubility of CXB. In particular, the solubilities of CXB in Cre-RH, Labrasol, Soluplus, Poloxamer 407, Ryoto sugar ester P-1670, and Ryoto sugar ester L-1695 solutions were 1434.7 ± 16.4, 1024.1 ± 27.9, 823.1 ± 30.7, 622.4 ± 22.0, 407.5 ± 0.6, and 329.3 ± 7.2 μg/mL, respectively. Among these, CXB solubility was highest at Cre-RH 5% with 1434.7 ± 16.4 μg/mL, which was 717.4 times higher than the CXB solubility of 2.0 ± 0.1 μg/mL without CXB solubilizer (Table 1). It has been reported that Cre-RH, Ryoto sugar ester P-1670 and L-1695 can greatly improve not only the solubility of poorly water-soluble drugs, but also the permeability of drugs [37,38]. In particular, Cre-RH has been reported to significantly increase the apparent coefficient of permeability by increasing the membrane fluidity in the gastrointestinal tract [37]. Because of this action, Cre-RH is known as an effective permeability modulator that improves drug absorption after oral administration. Cre-RH is also known to be a non-ionic surfactant with little toxicity [39]. In summary, Cre-RH was selected as the optimal excipient for the pharmaceutical formulation of CXB because of its excellent properties and the safety of Cre-RH as a solubilizer and absorption enhancer.

### 3.2. Physicochemical Characterizations of Solid Dispersion Granule and Tablet

#### 3.2.1. Formulation and Morphology

**Figure 1:**
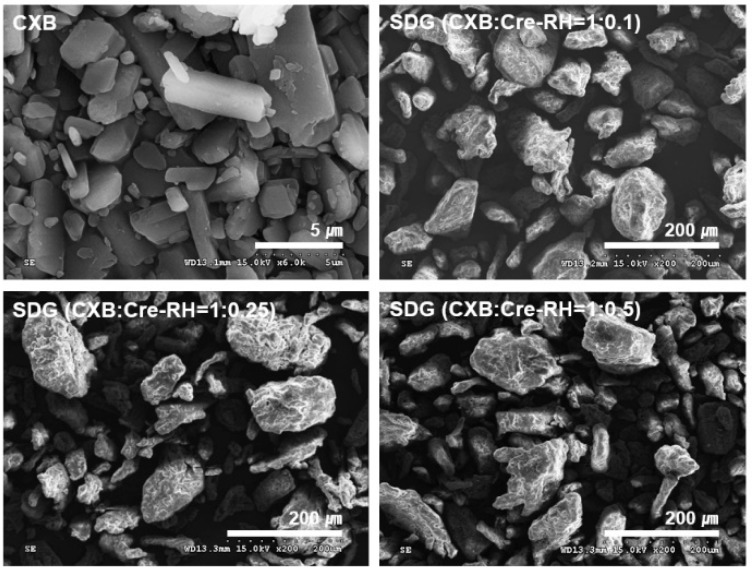
Scanning electron micrographs of CXB and solid dispersion granules (SDGs) with different weight ratios of CXB/Cre-RH. All the SDGs were 100–200 μm in size with irregular appearances.

Problems often arise during the process of manufacturing solid dosage forms, such as granules or tablets, using semi-solid materials, such as oil or lipids [38]. In this study, SDGs consisting of CXB, Cre-RH, HPMC, MCC, lactose, crospovidone, Aerosil, and talc were successfully prepared by the fluid-bed granulation process. The large-sized SDGs prepared by the granulation process have been shown to be advantageous in pharmaceutical processes, as micromeritic properties, such as flowability, compressibility, and packability are greatly improved compared with the case of fine powder [40]. Figure 1 shows the macroscopic morphology of CXB powder and the manufactured SDGs. CXB powder is a crystalline material that has a very large size of about 5 μm, whereas the SDGs produced are all 100–200 μm in size, indicating that a relatively large granule is produced. We also observed that the SDG particles were irregular and not smoother than the CXB powder. As shown in the photograph, the CXB powders are densely aggregated, whereas the SDG particles are uniformly spaced apart. Collectively, we have determined that the formulation presented in this study is suitable for the manufacture of large-sized granules with improved flow properties.

#### 3.2.2. Solid-State Characterization

**Figure 2:**
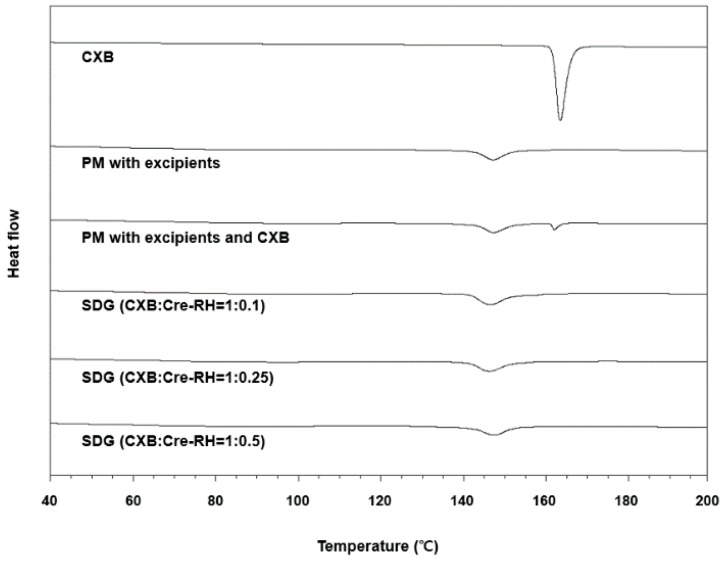
Differential scanning calorimeter patterns of CXB, physical mixture with excipients, physical mixture with excipients and CXB, and SDGs with different weight ratios of CXB/Cre-RH.

DSC thermograms of CXB, the physical mixture with excipients, and the physical mixture with excipient and CXB, and SDGs (CXB/Cre-RH = 1:0.1, 1:0.25, and 1:0.5) are shown in Figure 2. The DSC peak of CXB showed an endothermic pattern at 162–163 °C [41]. The distinct crystalline endothermic peak was also observed in the physical mixture with excipients, while the peaks disappeared in the SDG formulations. These results from the DSC crystallinity study indicate that CXB exists in an amorphous state within the prepared SDGs after the granulation process. These phenomena were the same as trends reported in previous studies: poorly water-soluble drugs are scattered or dissolved as an amorphous state in solid granule formulation, such as microcapsules and microspheres, prepared by spray-drying or fluid-bed granulation [31,38]. Therefore, we confirmed that CXB was moleculary dispersed or encapsulated within the granules, and excipients such as Cre-RH, microcrystalline cellulose, and lactose did not result in any changes in its amorphousness.

Flow characteristics of SDGs fabricated by fluid-bed granulation were verified by measuring the Hauser ratio (HR) and the Carr index (CI) [42]. Flow characteristics of powders are often presented according to HR and CI. Depending on their value, they are classified as excellent, good, fair, passable, poor, very poor, and non-flow [43]. The HR and CI values of CXB powder were 1.86 and 46.0, respectively, while the SDG values were very low at 1.14–1.23 and 12.8–19.2 (Table 2). Overall, the very poor flowability of CXB was found to be greatly improved as it was fabricated with granules containing CXB. This improvement in flowability was confirmed in the morphologies mentioned above. It was also observed by SEM that the cohesive and small-sized CXB particles were efficiently converted into large-sized, high density, and free-flowing granules by manufacturing processes. Thus, granules with good flowability will have several advantages in subsequent processes, such as tableting and capsule filling [44,45]. However, the high flowability of SDGs was found to gradually decrease with the increasing use of Cre-RH. The HR and CI values of SDG (CXB/Cre-RH = 1:0.1) were 1.14 and 12.8, respectively, which were good flow grades but fell into the category of fair grades of 1.23 and 19.2 for SDG (CXB/Cre-RH = 1:0.5) Therefore, the amount of Cre-RH in formulation should be carefully considered when applying the flowability and associated pharmaceutical processes in the manufacture of granules. The negative effect of Cre-RH on the flowability index (Cre-RH, 0.1–0.5) would not be problematic in subsequent processes and uses of the formulations. On the other hand, the hardness and friability of the SDT-containing SDGs were 5.3–5.8 Kg/cm^2^ and 0.45–0.51%, respectively, and there was no significant difference between the prepared formulations (Table 2). According to the USP guideline, less than 1% of the tablet’s friability is appropriate, but the results we obtained were much lower than this standard [46]. In addition, hardness above a reasonable level is known to be essential for maintaining product quality, and we have determined that values above 5.0 Kg/cm^2^ are sufficiently high. From these results, we have confirmed that the manufactured SDT is well-prepared with suitable tablets that can withstand the external influences during storage and use.

#### 3.2.3. Dissolution of CXB from SDG and SDT

**Figure 3:**
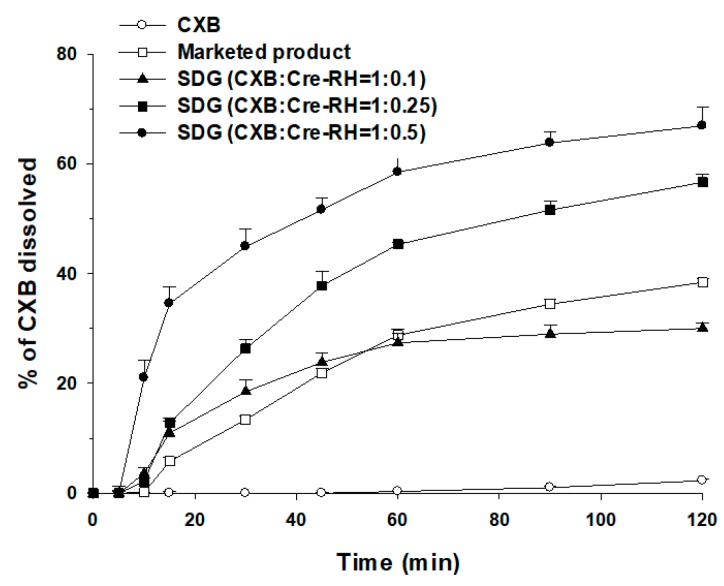
Dissolution profiles of CXB in pH 1.2 medium at 37.0 ± 0.5 °C from CXB, the marketed product (Celebrex^®^), and SDGs with different weight ratios of CXB/Cre-RH (*n* = 3).

CXB dissolution profiles of CXB powder, the marketed products, and SDGs (CXB:Cre-RH = 1:0.1, 1:0.25 and 1:0.5) are shown in Figure 3. The dissolution of CXB powder was 2.3 ± 0.2% at 120 min, which is almost no dissolution. However, SDGs containing Cre-RH showed a significant increase in dissolution rate compared with CXB powder. At 120 min, the CXB dissolutions of SDG (CXB/Cre-RH = 1:0.1), SDG (CXB/Cre-RH = 1:0.25), and SDG (CXB/Cre-RH = 1:0.5) increased to 30.0 ± 1.0, 56.6 ± 1.5, and 66.9 ± 3.5%, respectively, which were about 13, 24, and 29 times higher than those of CXB powder. The CXB dissolution of SDGs (CXB/Cre-RH = 1:0.25 and 1:0.5) is much higher than that of the marketed product (38.4 ± 0.7% at 120 min). These results can be explained by the fact that CXB is scattered or dissolved in an amorphous form in the matrix of granules due to the Cre-RH used as a solubilizer. Evidence for this result is a dissolution rate that increases with increasing Cre-RH. The dissolution rates of the granules (CXB/Cre-RH = 1:1 and 1:1.5) containing larger amounts of Cre-RH than those shown in this study were higher than those of the granules (CXB/Cre-RH = 1:0.1–0.5) (data not shown). Nevertheless, when an excess amount of Cre-RH is used, the fluid-bed granulation process does not proceed smoothly and the flowability of the produced granule is very low. Therefore, in this study, SDG (CXB/Cre-RH = 1:0.5), which shows the best flowability and dissolution and a smooth granulation process, was selected as the optimal formulation. The high dissolution profiles of CXB in aqueous media might be expected to contribute to the improvement in oral absorption.

**Figure 4:**
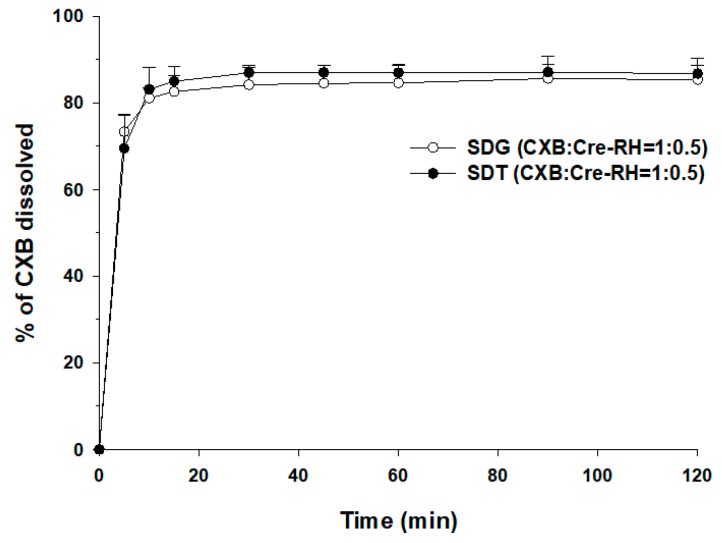
Dissolution profiles of CXB in pH 1.2 medium (sodium laurel sulfate (SLS) 0.5%) at 37.0 ± 0.5 °C from SDG and SDT (*n* = 3).

The dissolution profiles of SDG (CXB/Cre-RH = 1:0.5) and SDT (CXB/Cre-RH = 1:0.5) were obtained by tableting as the optimal granules were compared. Simultaneously, we observed the disintegration properties of SDTs in the vessels of the dissolution tester. The SDTs were completely disintegrated within 5 min. The dissolution profiles of the two formulations were almost similar (Figure 4). The dissolution profiles of the granules were frequently reported to change after the compression process [47,48]. The tablets having a hardness of 6.0 Kg/cm^2^ or less were used in this dissolution test (Figure 4). However, as mentioned in the literature [47,48], when the hardness of the tablets was more than 6.5 Kg/cm^2^, the dissolution profiles of CXB from the tablets were significantly reduced (data not shown). The dissolution profiles of the granules and tablets prepared in this study did not show any difference. In summary, the granules containing Cre-RH showed significantly improved CXB dissolution and retained an enhanced dissolution pattern even after the tableting process.

### 3.3. In Vivo Oral Pharmacokinetic Studies

**Figure 5:**
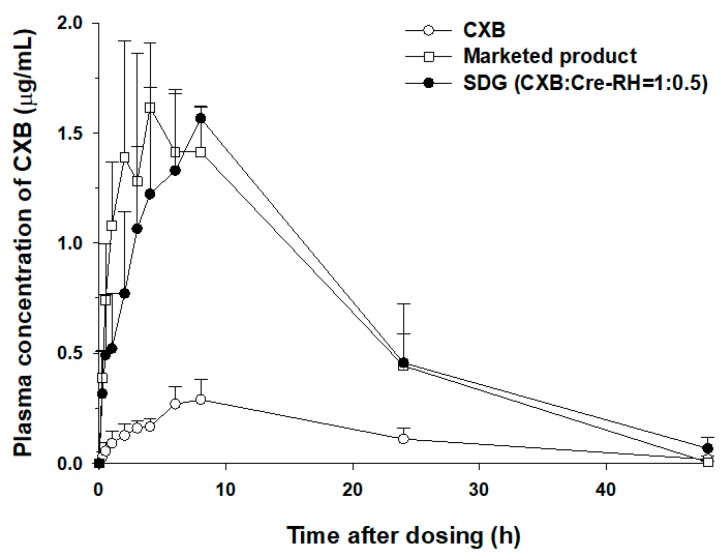
Plasma concentration versus time profiles of CXB in Sprague Dawley (SD) rats after oral administration of CXB, the marketed product (Celebrex^®^), and SDGs (CXB/Cre-RH = 1:0.5) at a dose of 5 mg/kg (*n* = 4–5).

Generally, a solid dispersion system has a molecularly dispersed drug in the polymeric matrix and shows a reduction in particle size, resulting in improved dissolution profiles and oral absorption [3,4]. In particular, Biopharmaceutical Classification System (BCS) class II drugs, such as CXB, can be significantly improved in dissolution and bioavailability when formulated with solid dispersion [49]. Consistently, these tendencies were observed in this study. Because the developed SDG (CXB/Cre-RH = 1:0.5) showed the highest dissolution profile among several formulations (Figure 3), further oral pharmacokinetic studies were conducted to investigate the possibility of increased oral absorption in rats. As shown in Figure 5, SDG-administered rats markedly showed high levels of blood concentration compared with CXB powder. The observed *C*_max_ and AUC_inf_ of SDG were 1.62 ± 0.07 μg/mL and 1954 ± 353 μg·min/mL, respectively, while those of the CXB powder were 0.33 ± 0.03 μg/mL and 423 ± 69 μg·min/mL, respectively (Table 3), indicating 4.9 and 4.6 times increases, respectively. The elimination half-life and MRT remained unchanged for the SDG formulation. In addition, the produced SDG formulation showed comparable AUC_inf_ compared with Celebrex^®^ (1954 ± 353 vs. 1853 ± 389 μg·min/mL). The enhanced oral *C*_max_ and relative bioavailability (BA) of CXB from the SDG formulation can be attributed to the increased solubility and/or high dissolution profiles resulting from its amorphous dispersion and melting in the SDG matrix. In addition, although our SDG formulation does not contain the anionic surfactant, unlike the marketed product (Celebrex^®^), it was comparable to Celebrex^®^ with respect to BA. As mentioned earlier, non-ionic surfactants are primarily considered in the use of pharmaceuticals in comparison with cationic and/or anionic surfactants due to various advantages in terms of efficiency, toxicity, biological compatibility, and biodegradability [20,24]. Moreover, the necessity of developing various formulations has been proposed to broaden the application of CXB, a high-dose and poorly water-soluble drug [50]. Because the marketed product is a hard gelatin capsule containing anionic SLS as a solubilizer to increase the oral absorption [25], the alternate method using SDGs and a tablet formulation containing non-ionic surfactants can be utilized to widen the usage of CXB through greater patient safety and compliance.

## 4. Conclusions

In this study, we developed a solid dispersion system to improve patient compliance and safety with respect to poorly water-soluble celecoxib (CXB). Solubilizer screening was carried out to select Cre-RH as the optimized solubilizer. Granules and tablets containing CXB and Cre-RH were prepared using the fluid-bed granulation and compression process. The morphology, crystallinity, flowability, dissolution, and pharmacokinetics of the prepared SDGs were evaluated. The SDG without anionic surfactant (SLS), a toxic material, proved to be able to significantly improve the solubility, dissolution, and bioavailability of CXB, compared with CXB powder. Moreover, the SDG without SLS exerted comparable dissolution and oral bioavailability compared with the marketed product. In conclusion, the SDG developed in this study could be used as an effective solid dosage form to simultaneously improve oral absorption, patient compliance, and the safety of CXB.

## Figures and Tables

**Table 1:** Saturated solubility and its relative ratio of celecoxib (CXB) in aqueous solutions with various solubilizers (5%) (*n* = 3).

Solubilizer	Solubility (μg/mL)	Relative Ratio (Versus CXB, -Fold)
No additive (only CXB)	2.0 ± 0.1	-
Cremophor RH40	1434.7 ± 16.4	717.4
Labrafac PG	8.0 ± 2.7	4.0
Labrafil M 1944 CS	284.3 ± 21.3	142.2
Labrasol	1024.1 ± 27.9	512.1
Plurol Oleique CC497	93.6 ± 12.2	46.8
Poloxamer 188	25.0 ± 13.3	12.5
Poloxamer 407	622.4 ± 22.0	311.2
Ryoto sugar ester L-1695	329.3 ± 7.2	164.7
Ryoto sugar ester P-1670	407.5 ± 0.6	203.8
Soluplus	823.1 ± 30.7	411.6
SPAN 80	290.6 ± 157.8	145.3
Transcutol HP	44.8 ± 27.1	22.4

**Table 2:** Physical properties of celecoxib powder, SDG and solid dispersion tablets (SDT).

Granules	Hausner Ratio	Carr’s Index (%)
Control (CXB)	1.86 ± 0.10	46.0 ± 2.83
SDG (CXB/Cre-RH = 1:0.1)	1.14 ± 0.01	12.8 ± 0.6
SDG (CXB/Cre-RH = 1:0.25)	1.17 ± 0.01	14.7 ± 0.5
SDG (CXB/Cre-RH = 1:0.5)	1.23 ± 0.01	19.2 ± 0.6
Tablets	Hardness (Kg/cm^2^)	Friability (%)
SDT (CXB/Cre-RH = 1:0.1)	5.8 ± 0.6	0.51
SDT (CXB/Cre-RH = 1:0.25)	5.3 ± 0.3	0.45
SDT (CXB/Cre-RH = 1:0.5)	5.6 ± 0.6	0.50

**Table 3:** Pharmacokinetic parameters of CXB in SD rats after oral administration of an equivalent dose (5 mg/kg) of CXB powder, the marketed product (Celebrex^®^), or SDGs (CXB/Cre-RH = 1:0.5) (*n* = 4–5).

Pharmacokinetic Parameters	CXB Powder	Marketed Product (Celebrex^®^)	SDG (CXB/Cre-RH = 1:0.5)
*T*_max_ (min)	450 ± 60	220 ± 35 *	450 ± 60
*C*_max_ (μg /mL)	0.33 ± 0.03	1.56 ± 0.31 *	1.62 ± 0.07 *
*T*_1/2_ (min)	520 ± 203	317 ± 60	523 ± 146
AUC_last_ (μg∙min/mL)	407 ± 54	1851 ± 386 *	1888 ± 289 *
AUC_inf_ (μg∙min/mL)	423 ± 69	1853 ± 389 *	1954 ± 353 *
MRT (min)	949 ± 215	720 ± 39	840 ± 232
Relative BA (%)	-	438	462

* *p* < 0.05, compared with the CXB powder.
